# Synthesis, Structural Characterization, and Antitumor Activity of a Ca(II) Coordination Polymer Based on 1,6-Naphthalenedisulfonate and 4,4′-Bipyridyl

**DOI:** 10.3390/ma6083547

**Published:** 2013-08-16

**Authors:** Xishi Tai, Wenhua Zhao

**Affiliations:** 1 College of Chemistry and Chemical Engineering, Weifang University, Weifang 261061, China; 2 Department of Chemistry, Qinghai Normal University, Xining 810008, Qinghai Province, China; E-Mail: zhaowenhua0214@163.com

**Keywords:** Ca(II) coordination polymer, synthesis, structural characterization, antitumor activity

## Abstract

A novel Ca(II) coordination polymer, [CaL(4,4′-bipyridyl)(H_2_O)_4_]*_n_* (L = 1,6-naphthalenedisulfonate), was synthesized by reaction of calcium perchlorate with 1,6-naphthalenedisulfonic acid disodium salt and 4,4′-bipyridyl in CH_3_CH_2_OH/H_2_O. It was characterized by elemental analysis, IR, molar conductivity and thermogravimetric analysis. X-ray crystallography reveals that the Ca(II) coordination polymer belongs to the orthorhombic system, with space group *P*2_1_2_1_2_1_. The geometry of the Ca(II) ion is a distorted CaNO_6_ pengonal bipyramid, arising from its coordination by four water molecules, one nitrogen atom of 4,4′-bipyridyl molecule, and two oxygen atoms from two L ligands. The complex molecules form a helical chain by self-assembly. The antitumor activity of 1,6-naphthalenedisulfonic acid disodium salt and the Ca(II) coordination polymer against human hepatoma *smmc-7721* cell line and human lung adenocarcinoma *A549* cell line reveals that the Ca(II) coordination polymer inhibits cell growth of human lung adenocarcinoma *A549* cell line with IC50 value of 27 μg/mL, and is more resistive to human lung adenocarcinoma *A549* cell line as compared to 1,6-naphthalenedisulfonic acid disodium salt.

## 1. Introduction

The last few decades, many studies have focused on the metal-organic hybrid materials constructed by *d*-block or *f*-block cations and organic ligands, because they have potential applications in catalysis [1], photoluminescence [2], gas storage [3], molecule-based magnetic materials [4] and biomedical materials [5]. In comparison to *d*-block cations, the coordination behavior and potential applications of alkaline earth metal complexes has remained largely an unexpored area [6]. As part of our group to explore the synthesis and properties of alkaline earth metal complexes, we have been exploring the preparation of metal-organic hybrid materials by combining alkaline earth metal ions and organic ligands containing multi-oxygen and nitrogen atoms [7,8,9,10,11]. In this paper, a new hybrid material, [CaL(4,4′-bipyridyl)(H_2_O)_4_] (L = 1,6-naphthalenedisulfonate) was synthesized and characterized by elemental analysis, IR, thermogravimetric analysis and X-ray structure analysis. On the basis that metal-organic frameworks can be used as delivery vehicles for drug molecules [12], the antitumor activity of 1,6-naphthalenedisulfonic acid disodium salt and its Ca(II) coordination polymer against human hepatoma *smmc-7721* cell line and human lung adenocarcinoma *A549* cell line also have been investigated.

## 2. Results and Discussion

### 2.1. Molar Conductivity 

The molar conductance value of the Ca(II) coordination polymer in methanol solution (1 × 10^−3^ mol L^−1^) is 8.9 S·cm^2^·mol^−1^, indicating that the Ca(II) coordination polymer is a nonelectrolyte [13].

### 2.2. IR Spectra

The IR spectrum of the Ca(II) coordination polymer exhibits ligand bands with the appropriate shifts due to complex formation. The *ν*(SO_3_^−^) vibrations at 1336 cm^−1^ and 1201 cm^−1^ in the free 1,6-naphthalenedisulfonate ligand shift to lower frequencies and are observed at 1301 cm^−1^ and 1186 cm^−1^ for the complex, indicating that the oxygen atoms of SO_3_^−^ coordinate to Ca(II) ions [14]. The IR spectrum of the Ca(II) coordination polymer displays peaks at 1219 cm^−1^, 800 cm^−1^ and 607 cm^−1^ which may be attributed to the *ν*(C=N) stretching of 4,4′-bipyridyl, showing that the nitrogen atoms of 4,4′-bipyridyl also take part in the coordination with calcium atom. In addition, at lower frequency the complex exhibits bands around 519 cm^−1^ and 417 cm^−1^ which may be assigned to *ν*(Ca–N) and *ν*(Ca–O) vibration.

### 2.3. Structure Description

**Figure 1:**
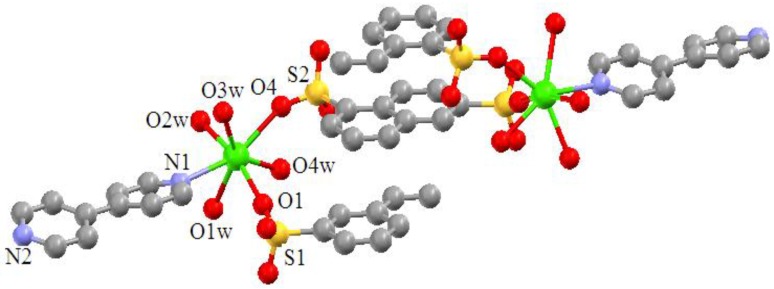
Coordination environment around the Ca(II) center in the coordination polymer.

**Figure 2:**
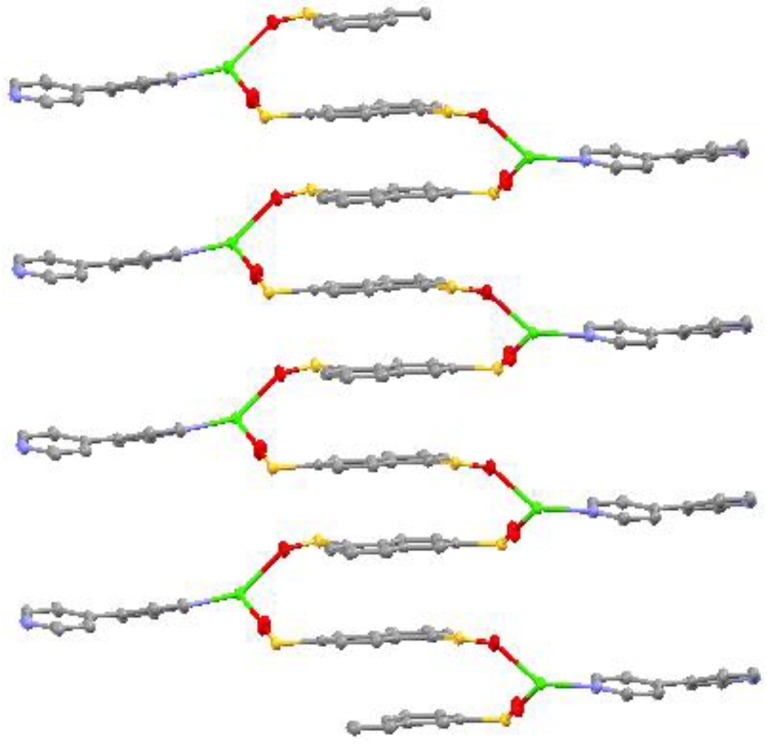
1D chained helical structure of the Ca(II) coordination polymer. The uncoordinated O atoms and the O atoms from coordinated water molecules were omitted for clarity.

**Figure 3:**
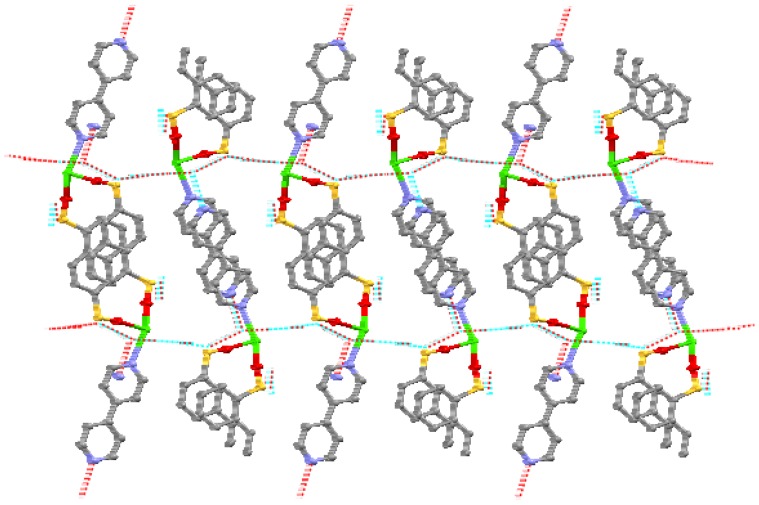
3D network structure of the Ca(II) coordination polymer.

A single-crystal X-ray crystallographic study reveals that the Ca(II) coordination polymer crystallizes in the space group *P*2_1_2_1_2_1_ with *Z* value of 4. The basic unit consists of one Ca(II) center with distorted pengonal bipyramidal geometry (Figure 1). The coordination environment around Ca(II) is afforded by two oxygen atoms (O1 and O4) from two 1,6-naphthalenedisulfonate ligands, four oxygen atoms (O1W, O2W, O3W and O4W) from four coordinated water molecules, and one nitrogen atom (N1) from 4,4′-bipyridyl ligand. The Ca(II) coordination polymer molecules from one-dimensional chained helical structures by the π–π interaction of the bridging ligand, 1,6-naphthalenedisulfonate (Figure 2). The helical chains are further connected by hydrogen bonds to form a three dimensional network structure (Figure 3). It is interesting that the 4,4′-bipyridyl only acts as monodentate ligand in the complex molecule. The distances of the Ca–O bonds are in the range of 2.2982(13) ~ 2.4896(14) Å, and that of Ca–N bond is 2.5934(15) Å, which are similar to the Ca–O bond lengths reported previously [15,16]. In addition, the formation of network benefits from the intermolecular and intramolecular hydrogen bonds (Table 1).

**Table 1:** Hydrogen-bond geometry (Å, °).

*D*–H···*A*	*D*–H	H···*A*	*D*···*A*	*D*–H···*A*
O(1W)–H(1A)···O(3W) ^i^	0.85	2.14	2.961(2)	163
O(1W)–H(1B)···O(3) ^ii^	0.84	2.02	2.803(2)	155
O(2W)–H(2A)···O(2) ^ii^	0.86	2.02	2.865(2)	170
O(2W)–H(2B)···O(3) ^iii^	0.85	1.95	2.798(19)	172
O(3W)–H(3A)···N(2) ^iv^	0.86	1.90	2.755(2)	172
O(3W)–H(3B)···O(2) ^v^	0.84	2.19	2.963(2)	154
O(4W)–H(4A)···O(6) ^i^	0.84	2.08	2.878(19)	157
O(4W)–H(4B)···O(5)	0.85	1.93	2.761(19)	166

Symmetry code: (i) −1 + *x*, *y*, *z*; (ii) 1 + *x*, *y*, *z*; (iii) 2 − *x*, −1/2 + *y*, ½ − *z*; (iv) 1 − *x*, −1/2 + *y*, ½ − *z*; and (v) ½ + *x*, 3/2 − *y*, 1 − *z*.

### 2.4. Thermogravimetric Analysis

The thermogravimetric analysis of Ca(II) coordination polymer was performed under air atmosphere. The TG measurement confirms that Ca(II) coordination polymer is thermally stable up to 180 °C. The TG curve indicates that Ca(II) coordination polymer starts to loose water molecules at *ca.* 180 °C and completes dehydration at *ca.* 200 °C. The mass loss is 12.36% in the range 180–200 °C, which corresponds to the loss of four water molecules. On further heating, the TG curve shows a continuous mass loss up to 650 °C due to decomposition of Ca(II) coordination polymer.

### 2.5. Antitumor Activity

The data of antitumor activities of Ca(II) coordination polymer and 1,6-naphthalenedisulfonic acid disodium salt are given in Table 2. Up to now, there have been no reports that calcium salts have antitumor activity. From Table 2, It can be seen that both Ca(II) coordination polymer and 1,6-naphthalenedisulfonic acid disodium salt exerted cytotoxic effect against human hepatoma *SMMC-7721* cells, and the antitumor effect of 1,6-naphthalenedisulfonic acid disodium salt is better than that of Ca(II) coordination polymer. However, Ca(II) coordination polymer has stronger cytotoxicity against human lung adenocarcinoma *A549* cells with lower IC_50_ (27 ± 1.2 μg/mL) than that of 1,6-naphthalenedisulfonic acid sodium. The result of molar conductivity of the Ca(II) coordination polymer shows that the Ca(II) coordination polymer is a nonelectrolyte, so we think that the antitumor activity of Ca(II) coordination polymer is due to the joint action of the ligand and the Ca(II).

**Table 2:** Antitumor activities of Ca(II) coordination polymer and 1,6-naphthalenedisulfonic acid disodium salt.

Compound	IC_50_ (μg/mL)	
	SMMC-7721	A549
1,6-naphthalenedisulfonic acid sodium	22 ± 1.7	–
Ca(II) complex	36 ± 2.9	27 ± 1.2

–: no antitumor activity.

## 3. Experimental Section

### 3.1. Materials and Methods

The 1,6-naphthalenedisulfonic acid disodium salt, Ca(ClO_4_)_2_·4H_2_O and 4,4′-bipyridyl ligands were purchased from Aldrich (St. Louis, MO, USA). All other regents used were analytical grade and used without further purification.

Elemental analysis (C, H, N) was carried out on a Elementar Vario III EL elemental analyzer (Hanau, Germany). Infrared spectra were recorded as KBr discs using a Nicolet AVATAR 360 FTIR spectrophotometer in the range 4000 cm^−1^ ~ 400 cm^−1^. Thermogravimetric analysis was performed on a Shimadzu PT-40 with heating rate programmed at 5 °C min^−1^. X-ray diffraction data of the Ca(II) complex was collected on a Bruker smart CCD diffractometer.

### 3.2. Synthesis of Ca(II) Coordination Polymer

A 5 mL methanol solution of 0.5 mmol (0.1555 g) Ca(ClO_4_)_2_·4H_2_O was added to a solution containing 0.5 mmol (0.1661 g) of 1,6-naphthalenedisulfonic acid disodium salt in 10 mL CH_3_CH_2_OH. The mixture was stirred for 2 h at refluxing temperature. Then 0.5 mmol (0.07809 g) 4,4′-bipyridyl was added to the above solution. The mixture was continuously stirred for 3 h at refluxing temperature. The white precipitates were collected by filtration. Then the white precipitates redissolved in MeOH, and the single crystal suitable for X-ray determination was obtained from methanol solution after 20 days by evaporation in air at room temperature. Elementary analysis: calcd for C_20_H_22_CaN_2_O_10_S_2_: C, 43.27; H, 3.97; 5.05%; found: C, 43.57; H, 3.58; and N, 4.73%. IR *ν*_max_(cm^−1^): *ν*(SO_3_^−^):1301 cm^−1^, 1186 cm^−1^, *ν*(C=N): 1219 cm^−1^, 800 cm^−1^ and 607 cm^−1^, *ν*(Ca–N): 519 cm^−1^ and *ν*(Ca–O): 417 cm^−1^.

### 3.3. X-ray Crystallography

Single crystal X-ray diffraction data were collected at 273(2) K on a Bruker smart CCD diffractometer using graphite-monochromatic Mo *Kα* radiation (*λ* = 0.71073 Å). The structure was solved by direct method and refined using a full-matrix least-squares technique against *F*^2^ with anisotropic displacement parameters for non-hydrogen atoms with the program SHELXL-97 [17]. All hydrogen atoms were placed at calculated positions using suitable riding models with isotropic displacement parameters derived from their carrier atoms. Molecular graphics were drawn with the program package SHELXTL-97 crystallographic software package [18].

A summary of crystal data and relevant refinement parameters for Ca(II) coordination polymer are as following: formula, C_20_H_22_CaN_2_O_10_S_2_; formula weight, 554.60; crystal system, orthorhombic; space group, *P*2_1_2_1_2_1_; *a* = 6.7583(5) Å; *b* = 14.8971(11) Å; *c* = 22.8915(17) Å; *α* = *β* = *γ* = 90°; *Z* = 4; *F*(000) = 1152; T = 273(2) K; *V* = 2304.7(3) Å^3^; calculated density, 1.598 μg·m^−3^; crystal size, 0.22 mm × 0.20 mm × 0.16 mm; *μ* = 0.514 mm^−1^; *S* = 1.022; limiting indices, −8 ≤ *h* ≤ 8, −18 ≤ *k* ≤ 15, −28 ≤ *l* ≤ 26; reflections collected/unique, 13037 and 4511; *R*_1_, *wR*_2_ [all data], 0.0235 and 0.0566; *R*_1_, *wR*_2_ [*I* > 2*σ*(*I*)], 0.0221 and 0.0557; *R*_int_ = 0.0176; goodness of fit on *F*^2^, 1.022; largest diff. peak and hole, 0.173 e·Å^−3^ and −0.213 e·Å^−3^. Selected bond lengths and angles are listed in Table 3.

**Table 3:** Selected bond lengths (Å) and angles (°) for Ca(II) coordination polymer.

Bonds	Bond length (Å)	Angles (°)
Ca(1)–O(1)	2.298(13)	–
Ca(1)–O(4W)	2.407(14)	–
Ca(1)–O(4)	2.426(13)	–
Ca(1)–N(1)	2.593(15)	–
Ca(1)–O(2W)	2.361(14)	–
Ca(1)–O(1W)	2.423(15)	–
Ca(1)–O(3W)	2.490(14)	–
O(1)–Ca(1)–O(2W)	–	164.41(6)
O(2W)–Ca(1)–O(4W)	–	92.61(6)
O(2W)–Ca(1)–O(1W)	–	79.81(5)
O(1)–Ca(1)–O(4)	–	98.95(5)
O(1)–Ca(1)–O(4W)	–	86.44(6)
O(1)–Ca(1)–O(1W)	–	85.04(6)
O(1W)–Ca(1)–O(4W)	–	73.07(6)
O(2W)–Ca(1)–O(4)	–	95.74(5)
O(4W)–Ca(1)–O(4)	–	74.14(5)
O(1)–Ca(1)–O(3W)	–	96.60(6)
O(4W)–Ca(1)–O(3W)	–	142.14(5)
O(4)–Ca(1)–O(3W)	–	68.10(4)
O(2W)–Ca(1)–N(1)	–	91.05(6)
O(1W)–Ca(1)–N(1)	–	73.61(5)
O(3W)–Ca(1)–N(1)	–	71.91(5)
O(1W)–Ca(1)–O(4)	–	146.63(5)
O(2W)–Ca(1)–O(3W)	–	93.68(5)
O(1W)–Ca(1)–O(3W)	–	144.75(5)
O(1)–Ca(1)–N(1)	–	81.10(5)
O(4W)–Ca(1)–N(1)	–	145.23(5)
O(4)–Ca(1)–N(1)	–	139.76(5)

### 3.4. Antitumor Activity

Human hepatoma *SMMC-7721* cells and human lung adenocarcinoma *A549* cells were propagated continuously in culture and grown in RPMI 1640 medium with 10% inactivated fetal calf serum and antibiotics. Cell harvested from exponential phase were seeded equivalently into 96 well plates and incubated for 24 h, then the solid compounds studied were added in a concentration gradient. The final concentrations were maintained at *c*/(μg mL^−1^) 5, 10, 20, 30, 40, 60 respectively. The plates were maintained at 37 °C in a humidified 5%CO_2_–90%N_2_–5%O_2_ atmosphere and incubated for 48 h, MTT solution was added, and the procedure described in [19] was then followed. The measurements of absorption of the solution concerned with the number of live cells were performed on spectrophotometer at 570 nm.

## 4. Conclusions

In summary, we have synthesized a new one-dimensional helical chained Ca(II) coordination polymer, [CaL(4,4′-bipyridyl)(H_2_O)_4_]*_n_*. The spectral properties, crystal structure and antitumor activity also have been investigated. Further investigation of the property and application of Ca(II) coordination polymer are currently in progress in our laboratory.

## Appendix

Crystallographic data for the structure reported in this paper has been deposited with the Cambridge Crystallographic Data Centre as supplementary publication No. CCDC 942222. Copy of the data can be obtained free of charge on application to CCDC, 12 Union Road, Cambridge CB2 1EZ, UK (Fax: +44-1223-336-033; E-Mail: deposit@ccdc.cam.ac.uk).
